# Exome Sequencing in 200 Intellectual Disability/Autistic Patients: New Candidates and Atypical Presentations

**DOI:** 10.3390/brainsci11070936

**Published:** 2021-07-16

**Authors:** Floriana Valentino, Lucia Pia Bruno, Gabriella Doddato, Annarita Giliberti, Rossella Tita, Sara Resciniti, Chiara Fallerini, Mirella Bruttini, Caterina Lo Rizzo, Maria Antonietta Mencarelli, Francesca Mari, Anna Maria Pinto, Francesca Fava, Margherita Baldassarri, Alessandra Fabbiani, Vittoria Lamacchia, Elisa Benetti, Kristina Zguro, Simone Furini, Alessandra Renieri, Francesca Ariani

**Affiliations:** 1 Medical Genetics, University of Siena, 53100 Siena, Italy; floriana.valentino@dbm.unisi.it (F.V.); lucia.bruno@dbm.unisi.it (L.P.B.); gabriella.doddato@dbm.unisi.it (G.D.); giliberti@student.unisi.it (A.G.); sara.resciniti@dbm.unisi.it (S.R.); fallerini2@unisi.it (C.F.); mirella.bruttini@dbm.unisi.it (M.B.); francesca.mari@dbm.unisi.it (F.M.); francesca.fava@dbm.unisi.it (F.F.); margherita.baldassarri@dbm.unisi.it (M.B.); alessandra.fabbiani@dbm.unisi.it (A.F.); vittoria.lamacchia@dbm.unisi.it (V.L.); alessandra.renieri@dbm.unisi.it (A.R.); 2 Med Biotech Hub and Competence Center, Department of Medical Biotechnologies, University of Siena, 53100 Siena, Italy; elisa.benetti@dbm.unisi.it (E.B.); kristina.zguro@student.unisi.it (K.Z.); simone.furini@dbm.unisi.it (S.F.); 3 Genetica Medica, Azienda Ospedaliera Universitaria Senese, 53100 Siena, Italy; rossella.tita@dbm.unisi.it (R.T.); lorizzo2@unisi.it (C.L.R.); mariaantonietta.mencarelli@unisi.it (M.A.M.); annamaria.pinto@dbm.unisi.it (A.M.P.)

**Keywords:** exome sequencing, intellectual disability, autism spectrum disorder

## Abstract

Intellectual disability (ID) and autism spectrum disorder (ASD) belong to neurodevelopmental disorders and occur in ~1% of the general population. Due to disease heterogeneity, identifying the etiology of ID and ASD remains challenging. Exome sequencing (ES) offers the opportunity to rapidly identify variants associated with these two entities that often co-exist. Here, we performed ES in a cohort of 200 patients: 84 with isolated ID and 116 with ID and ASD. We identified 41 pathogenic variants with a detection rate of 22% (43/200): 39% in ID patients (33/84) and 9% in ID/ASD patients (10/116). Most of the causative genes are genes responsible for well-established genetic syndromes that have not been recognized for atypical phenotypic presentations. Two genes emerged as new candidates: *CACNA2D1* and *GPR14*. In conclusion, this study reinforces the importance of ES in the diagnosis of ID/ASD and underlines that “reverse phenotyping” is fundamental to enlarge the phenotypic spectra associated with specific genes.

## 1. Introduction

Intellectual disability (ID) is characterised by significant limitations in intellectual functioning (reasoning, learning, problem-solving) and adaptive behaviour (conceptual, social, and practical skills) that originate before the age of 18 [1]. Affecting 1–3% of the world′s population, ID represents an important socio-economic problem in healthcare [2,3]. ID is characterized by limitations in cognitive functions that manifest as an intelligence quotient (IQ) below 70. ID may be “isolated” or “syndromic” when patients have peculiar facies, specific physical signs and/or an abnormal growth pattern [4].

Autism spectrum disorder (ASD) is characterized by deficient social interactions, poor or absent communication, repetitive behaviours, and apparently limited interests [5]. ASD generally becomes apparent after the first year of life and it has been reported in an increasing number (2.2–2.7%) of children, with boys four times more likely to be affected than girls [6,7]. From a clinical point of view, the autism spectrum disorder is subdivided into “syndromic” when a co-occurrence between autism (ASD) and dysmorphic features, also other somatic or neurobehavioral abnormalities could be observed [8].

ID and ASD often co-exist and identifying the etiology of the two conditions remains challenging due to disease heterogeneity. Accurate clinical, as well as molecular diagnoses are essential for a deeper understanding of the pathogenesis of these conditions and for devising tailored treatments [4]. Until the advent of the first next generation sequencing (NGS) platforms, a large fraction of cases remained not diagnosed, with many families undergoing a “diagnostic odyssey” [9]. The introduction of exome or genome sequencing (ES/GS) has significantly improved diagnostic rates in individuals with suspected ID/ASD genetic disorders refractory to conventional diagnostic testing [10].

In the present study, ES was generated for a total of 200 individuals (84 ID and 116 ID/ASD patients). Pathogenic or likely pathogenic (P/LP) variants were found in 43 individuals (22%), with 45 variants of uncertain significance in an additional 20% (40/200). Our data strongly support the value of large-scale sequencing, especially ES within proband-parent trios, as an effective first-choice diagnostic tool.

## 2. Materials and Methods

### 2.1. Selection of Patients and DNA Samples’ Preparation

Genetic counselling was carried out to evaluate each patient′s personal and familial history. Parents provided and signed a written informed consent at the Medical Genetics department of the University of Siena, Italy, for exome sequencing analysis, clinical data usage, and the use of DNA samples from the tested individuals for both research and diagnosis purposes. We analysed a total of 200 patients affected by ID and ID/ASD (84 with ID and 116 with ID and ASD) collected from January 2019 until the end of March 2021.

Genomic DNA from the parents was isolated from EDTA peripheral blood samples using MagCore HF16 (Diatech Lab Line, Jesi, Ancona, Italy) according to the manufacturer’s instructions.

### 2.2. Exome Sequencing

Sample preparation was performed following the Illumina DNA Prep with Enrichment manufacturer protocol. A bead-based transposome complex is used to perform tagmentation, a process that fragments the genomic DNA and then tags it with adapter sequences in one step. After saturation with input DNA, the bead-based transposome complex fragments a set number of DNA molecules. This fragmentation provides flexibility to use a wide DNA input range to generate normalized libraries with a consistent tight fragment size distribution. Then a limited-cycle PCR adds adapter sequences to the ends of a DNA fragment. A subsequent target enrichment workflow is then applied. Following pooling, the double-stranded DNA libraries are denatured and biotinylated. Illumina Exome Panel v1.2 (CEX) probes are hybridized to the denatured library fragments. Then Streptavidin Magnetic Beads (SMB) capture the targeted library fragments within the regions of interest. Then the indexed libraries are eluted from beads and further amplified before sequencing. The exome sequencing analysis was performed on the Illumina *NovaSeq6000 System* (Illumina San Diego, CA, USA) according to the NovaSeq6000 System Guide. Reads were mapped against the hg19 reference genome using the Burrow-Wheeler aligner BWA [11]. Variant calling was obtained using an in-house pipeline which takes advantage of the GATK Best Practices workflow [12].

Prioritization of the variants was obtained excluding polymorphisms (minor allele frequency, MAF <0.01), synonymous variants, variants classified as benign or likely benign. Frameshift, stopgain, and splice site variants were prioritized as pathogenic. Missense variants were predicted to be damaging by CADD-Phred prediction tools. The potential impact of variants on splicing was evaluated using Alamut^®^ Visual software—version 2.11–0 (Interac-tive Biosoftware, Rouen, France), which employs five different algorithms: SpliceSiteFinder-like, MaxEntScan, NNSPLICE, GeneSplicer, and HumanSplicingFinder.

The following public databases were used for the interpretation of the variants: ClinVar (https://www.ncbi.nlm.nih.gov/clinvar/, accessed on 9 June 2021), LOVD (https://databases.lovd.nl/shared/genes, accessed on 9 June 2021), the Human Genome Mutation Database (HGMD, http://www.hgmd.cf.ac.uk/ac/index.php3, accessed on 9 June 2021).

## 3. Results

### 3.1. Clinical Characteristics of Patients

**Figure 1:**
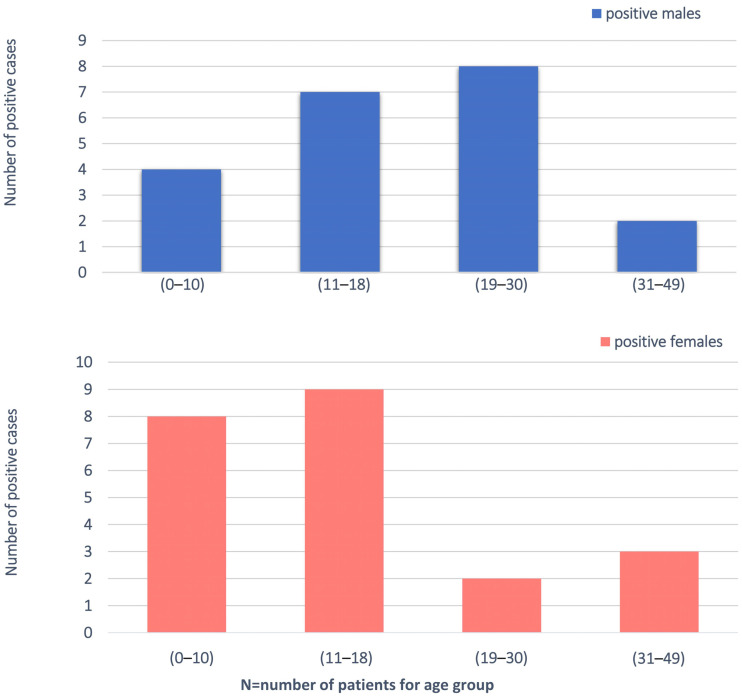
Demographic analysis of the cohort, filtered by age and gender.

We enrolled 200 families (574 individuals total) with at least one proband with an unexplained diagnosis of ASD/ID-related phenotype. In particular, 181 families had one affected proband and 4 families had two affected probands. ES was sequenced to an average depth of 100×, respectively, with ~94% of bases covered ≥ 20×. The study population had a mean age of 15 years and was 64% male (128/200). We divided into four groups on the basis of the age (0–10; 11–18; 19–30; 31–49) and gender (Figure 1). All individuals displayed ID, 58% (116/200) associated with ASD. The totality of the patients had been subjected to genetic testing prior to enrolment in this study. Our ID and ID/ASD cohort was characterized by the presence of additional associated clinical findings for 90% of the patients (179/200). These included epilepsy (*n* = 41/200, 20%), hypotonia (*n* = 10/200, 5%), and MRI abnormalities observed in 13 patients. Craniofacial dysmorphisms (*n* = 106/200, 53%) were found in 53% of the cases. The clinical descriptions of the 200 patients are summarized in Table S1.

### 3.2. P/LP Variants Identified by ES

ES was performed in 200 probands with ASD and/or ID and 41 different pathogenic/likely pathogenic (P/LP) variants were identified with a detection rate of 22% (Table S2). Clinical features of patients with pathogenic variants in disease genes were described in Table 1.

Variants were classified based on frequency, mutation category, literature, and databases such as ClinVar. Affected individuals were categorized based on the number of parents that were sequenced along with the proband(s): proband-parent trios (184); duos with one parent (6); and proband-only singletons (10). Most P/LP variants were missense variants (15/200; 8%), while 5% (9/200) were nonsense, 7% (13/200) frameshift and 2% (4/200) splicing variants. Most (62%) P/LP variations occurred de novo, while 29% of individuals inherited P/LP variants as heterozygotes or homozygotes. Four out of 200 (2%) participants who harboured a P/LP result were sequenced with one or no biological parent and thus have unknown inheritance.

Mutations in the following genes were found in patients suffering from ID: ADNP, AP4M1, ATP1A3, BSCL2, CACNA1A, CSNK2B, CTU2, DEPDC5, DYRK1A, EFTUD2, HK1, IQSEC2, KANSL1, KIF1A, KMT2A, MBOAT7, MED13L, MMACHC, POGZ, PTPN11, RHOBTB2, SHANK3, SPG7, SPTBN2, TBCE, TUBA1A, WDR45, WFS1. In patients presenting autism and ID we found pathogenic variants in: BCOR, DDX3X, FGFR3, KCNQ3, SHANK3, SYNGAP1, TREX1, UPF3B, WFS1 (Table S2).

Two de novo P/LP variants were found in the new candidate genes: *CACNA2D1* and *GPR14* (Table S3), and the corresponding clinical pictures were reported in Table 2.

### 3.3. Uncertain Variants Identified by ES

We further reported 45 uncertain variants including in this number the variants that are currently considered to have an uncertain significance in the databases and other missense variants that have not been previously described in the scientific literature (Table S4). The effect on the encoded mutated proteins has been predicted using CADD (combined depletion annotation depletion). In our study the majority of uncertain and of P/LP variants fall in genes that play a role in the axon guidance and in the neurodevelopment processes (https://reactome.org/PathwayBrowser/#/, accessed on 6 July 2021).

## 4. Discussion

This study emphasizes the clinical diagnostic relevance of ES in patients with ID and/or autism with additional clinical features. In particular, in a cohort of 200 patients, we reached a diagnostic yield of 22% (43/200) with a higher rate in ID patients (33/84; 39%) with respect to ID and ASD patients (10/116; 9%). The diagnostic yield is lower with respect to other studies that employ ES in neurodevelopmental disorder (30–43%) [13]. ES has technological limitations, including the inability to detect noncoding variants, copy number variants (CNVs), epigenetic changes, and trinucleotide repeat expansion [14]. In our cohort, 30% of cases have not been screened for CNVs and this could have underestimated the presence of other pathogenic genetic alterations, in particular in patients with ASD. Until recently, whole genome chromosomal microarray was recommended as a first-tier clinical genetic test for detecting disease-causing CNVs in individuals with ASD [15,16,17].

Most of the previous diagnoses failed for the atypical phenotypic presentation of well-established genetic syndromes. The *DDX3X* mutated patient (#11) shows Rett-like spectrum features with typical hand-washing stereotypes and was initially screened for mutations in *MECP2, FOXG1* and *CDKL5* genes [18,19,20,21]. Differently, a hundred patients are reported in literature mutated in *DDX3X* with various clinical features including hypotonia, movement disorder, behavioural problems, corpus callosum hypoplasia and epilepsy [22,23]. Another atypical clinical picture was manifested by a patient bearing the mutation c.688C > T (p.(Arg230Cys)) in *KCNQ3*, who did not suffer from any status epilepticus but showing ID, autism, stereotypies, aggressiveness, bladder anomalies; he also presented craniofacial dysmorphisms (patient #18). *KCNQ3* pathogenic alterations are generally linked to the occurrence of seizures but recently patients with no EEG abnormalities have been described [24]. *KIF1A* gene was found altered in three unrelated cases with different phenotypic presentations (patients #19, #20, #21). *KIF1A* mutations cause NESCAV syndrome (NESCAVS), a neurodegenerative disorder characterized by global developmental delay, progressive spasticity, ID, speech delay, learning disabilities and/or behavioural abnormalities [25]. The mutation c.37C > T (p.(Arg13Cys)) in *KIF1A* was found in patient #19 with spastic paraparesis, behaviour disorder, slight enlargement of the interfolial spaces of the cerebellar hemispheres, hypertone and no craniofacial dysmorphisms. In particular the same mutation c.914C > T (p.(Pro305Leu)) was shown in two different patients (#20, #21). One of these, presented language delay, cerebellar and vermis atrophy, psychomotor delay, brain abnormalities, hypertrichosis, bilateral clinodactyly, and facial dysmorphisms (patient #20). The other showed ataxia, spastic paraparesis, angioma, nystagmus, and seizures (patient #21). Mutations in *POGZ* are associated with the White-Sutton syndrome, which is a neurodevelopmental disorder characterized by delayed psychomotor development and a characteristic constellation of dysmorphic facial features [26]. Additional features may include hypotonia, sensorineural hearing impairment, visual defects, joint laxity, and gastrointestinal difficulties [27]. The pathogenic variant c.1180_1181del (p.(Met394Valfs*9)) was carried by two siblings and their mother (#26, #27, #28). The sister exhibited craniofacial dysmorphisms and ID; she also showed hyperactivity, blepharophimosis, brachydactyly, nail hypoplasia, kidney abnormalities and language delay as additional clinical signs (patient #26). The brother was affected by ID, hypotonia, obesity and had some craniofacial dysmorphisms (patient #27). Their mother instead displayed ID, microcephaly, brachydactyly, and nail hypoplasia (patient #28). Another example, *SHANK3* was found altered in three unrelated patients (#31, #32, #33). Mutations in *SHANK3* cause Phelan-McDermid syndrome, a developmental disorder with variable features including neonatal hypotonia, global developmental delay, absent to severely delayed speech, autistic behaviour, and minor dysmorphic features [28,29,30]. One of the three mutated patients did not show neither autism nor dysmorphic features and was initially classified as a “non-syndromic” ID case.

The following new candidate genes for ID/ASD have emerged: *CACNA2D1* and *GPR14*. They all show de novo truncating variants (patients #44–#45). *CACNA2D1* encodes the alpha-2/delta subunit of skeletal muscle and brain voltage-dependent calcium channels [31]. A genomic aberration affecting the *CACNA2D1* gene has been previously characterized in patients with epilepsy and ID, pinpointing the gene as an interesting candidate gene for these clinical features [32]. Mice-bearing point mutations in the *CACNA2D1* gene have an abnormal central nervous system synaptic transmission [33]. *GPR14* gene, encoding the orphan G protein-coupled receptor 14 for Urotensin II, is widely expressed in the brain and spinal cord [34]. We found 45 variants of unknown significance (VUS) in 40 patients. These variants are 22/84 (26%) in ID patients and 18/116 (16%) in ID/ASD patients. These variants are mostly missense 42/45 (93%) and CADD ≥ 25 in 20/42 (48%) of cases. With increased knowledge over time, exome reanalysis may change the clinical interpretation of a VUS. Thus, it is important to list all the VUS, analyse them periodically and write a report in case of changes to provide a timely response for patients and families. An accurate molecular diagnosis allows for precise genetic counselling and has the potential to change clinical management.

## 5. Conclusion

ES was able to avoid a sort of “diagnostic odyssey” for a significant fraction of families consisting in the step-by-step application of the traditional genetic methods. ES revealed atypical phenotypic presentations and new candidate genes for ID/ASD. Further studies are needed to better characterize the contribution of new candidates and to show how their haploinsufficiency can determine ID/ASD.

## Figures and Tables

**Table 1:** Clinical features of patients with pathogenic variants in disease genes.

Patient	Gene	Variant (HGVS)	Protein (HGVS)	Gender	Age (Years)	ID/ASD	Craniofacial Dysmorphisms	Additional Clinical Signs	OMIM Phenotype (n°)
#1	*ADNP*	c.2314dup	p.(Thr772Asnfs *16)	Male	7	ID	Yes (Plagiocephaly, prominent forehead, sparse eyebrows, broad nasal bridge, small ears, uplifted earlobes, conical teeth)	Bilateral clinodactyly of the 5th toe, supernumerary nipple, epilepsy, heteroaggressive behaviour	Helsmoortel-van der Aa syndrome (#615873)
#2	*AP4M1*	c.1257_1282del c.1317G > A	p.(Val421Alafs *98) p.(Trp439 *)	Female	18	ID	Yes (microcephaly, triangular face, widow’s peak, thick eyebrows, large nose, flat philtrum, thin lips, high vaulted and narrow palate)	Short stature, clinodactyly of the fifth finger, laryngomalacia, splenium thinning, epilepsy, hearing loss, flat foot, stereotypies, epilepsy, speech defect	Spastic paraplegia 50, autosomal recessive (#612936)
#3	*ATP1A3*	c.2116G > A	p.(Gly706Arg)	Female	22	ID	No	Epilepsy, ataxia, limited speech	Alternating hemiplegia of childhood 2 (#614820)/CAPOS syndrome (#601338)/Dystonia-12 (#128235)
#4	*BCOR*	c.776C > T	p.(Ser259Leu)	Male	14	ID and ASD	Yes (low hairline, periorbital swelling, big mouth, full lips)	Stereotypies	Microphthalmia, syndromic, 2 (#300166)
#5	*BSCL2*	c.856C > T	p.(Arg286 *)	Female	22	ID	Yes (elongated facies, prominent ears, high nasal bridge, open mouth and protruded tongue)	Dystonia, myoclonia, drug-resistant epilepsy, language delay, psychomotor delay, bruxism, hypertrichosis	Encephalopathy, progressive, with or without lipodystrophy (#615924)
#6	*BSCL2*	c.856C > T	p.(Arg286 *)	Female	16	ID	Yes (facies with coarse features, synophria, bulbous nose, large ears, large mouth)	Dystonia, myoclonia, hypertrichosis, language delay, drug-resistant epilepsy, psychomotor delay	Encephalopathy, progressive, with or without lipodystrophy (#615924)
#7	*CACNA1A*	c.4055G > A	p.(Arg1352Gln)	Male	14	ID	Yes (Craniosynostosis, long filter, long eyelashes, thin upper lip)	Stereotypies, alternating hemiplegia, language delay	Developmental and epileptic encephalopathy 42 (#617106); Episodic ataxia, type 2 (#108500); Migraine, familial hemiplegic, 1 (#141500); Migraine, familial hemiplegic, 1, with progressive cerebellar ataxia (#141500); Spinocerebellar ataxia 6 (#183086)
#8	*CSNK2B*	c.170del	p.(Glu57Glyfs *15)	Female	20	ID	Yes (Open mouth, protruding tongue, narrow palate, dental anomalies)	Epilepsy, hypotonia, developmental delay, absent language	Poirier-Bienvenu neurodevelopmental syndrome (#618732)
#9	*CTU2*	c.881C > A	p.(Ser294 *)	Female	22	ID	Yes (broad eyebrows, low anterior and posterior hairline, forehead hypertrichosis, prominent ears, eye asymmetry, large nose with wide nostrils, large mouth, dental anomalies)	Upper limb hypotonia, lower limb hypertonia, recurrent infections, thinning corpus callosum, growth retardation, pectus excavatum, clinodactyly of the 5th fingers.	Microcephaly, facial dysmorphism, renal agenesis, and ambiguous genitalia syndrome (#617057)
#10	*DEPDC5*	c.1453C > T	p.(Arg485 *)	Male	11	ID	Yes (low hairline, flattened nasal bridge, bulbous nasal tip, anteverted nostrils, full lips, small and widely spaced teeth, anteverted ears)	Epilepsy, language delay	Epilepsy, familial focal, with variable foci 1 (#604364)
#11	*DDX3X*	c.976C > T	p.(Arg326Cys)	Female	12	ID and ASD	Yes (Microcephaly, long face, smooth and long philtrum, strabismus, up slanting palpebral fissures)	Developmental delay, stereotypic hand movements, hypotonia, bruxism, sialorrhea, corpus callosum hypoplasia	Intellectual developmental disorder, X-linked, syndrome, Snijders Blok type (#300958)
#12	*DYRK1A*	c.1669C > T	p.(Gln557 *)	Male	16	ID	Yes (Microcephaly, narrow forehead, frontal bossing, depressed nasal bridge, short philtrum, prognathism)	Hypotonia, epilepsy, stereotypies, absent language	Mental retardation, autosomal dominant 7 (#614104)
#13	*EFTUD2*	c.702 + 1G > A	NA	Female	22	ID	Yes (prominent columella, sloping forehead, high and scattered eyebrows, deeply set eyes, nose with anteverted nostrils, narrow palate, prominent incisors, prominent ears)	Hypertrichosis, small hands and feet, joint hyperextensivity	Mandibulofacial dysostosis, Guion-Almeida type (#603892)
#14	*FGFR3*	c.749C > G	p.(Pro250Arg)	Female	4	ID and ASD	Yes (craniosynostosis, flat forehead, down slanting palpebral fissures, high nasal bridge)	Language delay, hyperchromic and hypochromic spots	Muenke syndrome (#602849) CATSHL syndrome (#610474)
#15	*HK1*	c.1367C > T	p.(Thr456Met)	Male	16	ID	Yes (proptosis, long eyelashes, synophria, narrow palate, dental anomalies, small mouth, and full lips)	Bilateral cryptorchidism, psychomotor delay, poor vision, ventricular system dilation, immature hippocampal structures, spastic paraparesis, epilepsy, hypothonia, absent language	Neurodevelopmental disorder with visual defects and brain anomalies (#618547)
#16	*IQSEC2*	c.3780del	p.(Gln1261Serfs *136)	Female	22	ID	No	Strabismus, cerebral atrophy, sialorrhea, motor problems, bruxism	Mental retardation, X-linked 1/78 (#309530)
#17	*KANSL1*	c.985_986del	p.(Leu329Glufs *22)	Female	6	ID	Yes (cleft palate, deeply set eyes, high nasal bridge, bulbous nasal tip)	Language delay, epilepsy, aggressiveness	Koolen-De Vries syndrome (#610443)
#18	*KCNQ3*	c.688C > T	p.(Arg230Cys)	Male	39	ID and ASD	Yes (triangular facies, high nasal bridge, bulbous nasal tip, open mouth, micrognathia, narrow and downturned eyelids)	Stereotypies, aggressiveness, bladder anomalies	Seizures, benign neonatal, 2 (#121201)
#19	*KIF1A*	c.37C > T	p.(Arg13Cys)	Female	16	ID	No	Spastic paraparesis, behaviour disorder, slight enlargement of the interfolial spaces of the cerebellar hemispheres, hypertone	Spastic paraplegia 30, autosomal dominant (#610357)
#20	*KIF1A*	c.914C > T	p.(Pro305Leu)	Female	10	ID	Yes (sparse eyebrows, small nose, thin upper lip, dental anomalies, chubby cheeks)	Language delay, cerebellar and worm atrophy, psychomotor delay, brain abnormalities, hypertrichosis, bilateral clinodactyly of the second/third/fourth/fifth toes	Spastic paraplegia 30, autosomal dominant (#610357)
#21	*KIF1A*	c.914C > T	p.(Pro305Leu)	Male	49	ID	No	Ataxia, spastic paraparesis, angioma, nystagmus, epilepsy	Spastic paraplegia 30, autosomal dominant (#610357)
#22	*KMT2A*	c.5256delA	p.(Ala1753Profs*70)	Male	16	ID	Yes (thick eyebrows, narrow and short eyelids, depressed helix, nose with bulbous tip, thick lips)	Hypertrichosis of the legs, arms and lumbar region, large hands, short fifth ray of the foot	Wiedemann-Steiner syndrome (#605130)
#23	*MBOAT7*	c.477C > G	p.(Tyr159 *)	Female	2	ID	No	Motor stereotypies, epilepsy, thinning of the corpus callosum, ventricular enlargement	Mental retardation, autosomal recessive 57 (#617188)
#24	*MED13L*	c.72 + 1G > T	NA	Female	49	ID	Yes (sparse eyebrows, hypertelorism, narrow eyelids, gingival hypertrophy)	Syndactyly of the second and third toes, small feet, hypertrophy of the limbs and truncal obesity, strabismus	Mental retardation and distinctive facial features with or without cardiac defects (#616789)
#25	*MMACHC*	c.440G > C	p.(Gly147Ala)	Male	12	ID	Yes (synophria, horizontal eyebrows, wide nasal tip, anteverted nostrils, long filter, dental anomalies)	Spastic paraparesis, language delay, polyneuropathy	Methylmalonic aciduria and homocystinuria, cblC type (#277400)
#26	*POGZ*	c.1180_1181del	p.(Met394Valfs *9)	Female	6	ID	Yes (microcephaly, deeply set eyes, nose with bulbous tip, anteverted nostrils, full lips)	Hyperactivity, blepharophimosis, brachydactyly, nail hypoplasia, kidney abnormalities, language delay	White-Sutton syndrome (#616364)
#27	*POGZ*	c.1180_1181del	p.(Met394Valfs *9)	Male	12	ID	Yes (narrow bitemporal diameter, narrow and upward eyelid rims, deep philtrum, progatism, exaggerated Cupid′s bow, buccal rim pointing downwards, uplifted ear lobe)	Hypotonia, obesity	White-Sutton syndrome (#616364)
#28	*POGZ*	c.1180_1181del	p.(Met394Valfs *9)	Female	44	ID	No	Microcephaly, brachydactyly, nail hypoplasia	White-Sutton syndrome (#616364)
#29	*PTPN11*	c.1471C > A	p.(Pro491Thr)	Male	2	ID	Yes (high forehead, low-set ears with large, downward-pointing auricles, down-slanting eyelids, broad nasal tip, long and thick filter, exaggerated Cupid′s bow)	Café au lait spots in the thoracic and lumbar region, nail hypoplasia of the fifth toe, neurodevelopmental delay, cryptorchidism	Noonan syndrome 1 (#163950)
#30	*RHOBTB2*	c.1382G > A	p.(Arg461His)	Male	18	ID	Yes (microcephaly, thick eyebrows, deeply set eyes, square chin)	epilepsy, thinning corpus callosum, language delay, cold extremities, cerebellar atrophy, muscle hypotrophy	Developmental and epileptic encephalopathy 64 (# 618004)
#31	*SHANK3*	c.2717_2718dup	p.(Ser907Alafs *3)	Male	45	ID and ASD	Yes (alopecia in the fronto-temporal region, depressed ocular region, thin upper lip)	Absent language, obsessive behaviour, tapered fingers with widening of the intermediate interphalangeal joints, nail dystrophy, clinodactyly of the third toe, stereotypies, mouth chewing automatisms	Phelan-McDermid syndrome (#606232)
#32	*SHANK3*	c.2313 + 1G > A	NA	Male	21	ID and ASD	No	epilepsy, psychomotor delay	Phelan-McDermid syndrome (#606232)
#33	*SHANK3*	c.3250_3253del	p.(Leu1084Cysfs *9)	Male	18	ID	No	Language delay, psychomotor delay, manual stereotypies, increased tolerance to pain	Phelan-McDermid syndrome (#606232)
#34	*SPG7*	c.233T > A	p.(Leu78 *)	Female	10	ID	Yes (high forehead, spaced teeth, pursed lips attitude)	Epilepsy, neurodevelopmental regression, stereotypies, syndactyly	Spastic paraplegia 7, autosomal recessive (#607259)
#35	*SPTBN2*	c.1310G > A	p.(Arg437Gln)	Male	17	ID	Yes (sloping forehead, prognathism)	Strabismus, hyperchromic spot in the thoracic area, cerebellar atrophy, language delay, ataxia	Spinocerebellar ataxia 5 (#600224)
#36	*SYNGAP1*	c.2337–1G > A	NA	female	26	ID and ASD	No	Stereotypies, self-harm, cold extremities, sphincter control not acquired, language regression	Mental retardation, autosomal dominant 5 (#612621)
#37	*TBCE*	c.464T > A c.134dupA	p.(Ile155Asn) p.(Arg46Glufs *5)	Male	21	ID	Yes (bulbous nasal tip, full lips, spaced teeth)	Scoliosis, sialorrhea, optic atrophy, psychomotor delay, epilepsy, spastic paraparesis, absent language.	Encephalopathy, progressive, with amyotrophy and optic atrophy (#617207)
#38	*TREX1*	c.558_573del	p.(Phe186Leufs *24)	Male	10	ID and ASD	No	Gastroesophageal reflux, sphincter control not acquired, stereotyped behaviour	Aicardi-Goutieres syndrome 1 dominant and recessive (#225750)
#39	*TUBA1A*	c.352G > A	p.(Val118Met)	Male	9	ID	Yes (advanced hairline, long and thick eyebrows, anteverted nostrils, long filter, thin upper lip)	Hypoplasia of the cerebellar vermis, thinned corpus callosum, cerebellar asymmetry, angioma, hypertrichosis, epilepsy, apraxia, ataxia, and psychomotor delay	Lissencephaly 3 (#611603)
#40	*UPF3B*	c.1288C > T	p.(Arg430 *)	Male	7	ID and ASD	Yes (Wide forehead, arched eyebrows, deeply set eyes, pointed chin)	Limited speech, neurodevelopmental delay	Mental retardation, X-linked syndromic 14 (#300676)
#41	*WDR45*	c.66del	p.(Cys23Alafs *15)	Female	20	ID	Yes (thick eyebrows, prominent upper arch, hyperemic gums, high palate)	Limited speech, scoliosis, locomotor impairment, manual stereotypies, tapered fingers	Neurodegeneration with brain iron accumulation 5 (#300894)
#42	*WFS1*	c.124C > T	p.(Arg42 *)	Male	6	ID and ASD	No	Language delay, oppositional and provocative behaviour	Wolfram-like syndrome, autosomal dominant (#614296)
#43	*WFS1*	c.1230_1233del	p.(Leu412Serfs *29)	Female	14	ID	Yes (microcephaly, synophria, long eyelashes, bulbous nasal tip)	Hypertrichosis, drug-resistant seizures, spastic tetraparesis, renal failure.	Wolfram-like syndrome, autosomal dominant (#614296)

* means change in a stop codon.

**Table 2:** Clinical features of patients with pathogenic variants in the new candidate genes.

Patient	Gene	Variant (HGVS)	Protein (HGVS)	Gender	Age (Years)	ID/ASD	Craniofacial Dysmorphisms	Additional Clinical Signs
#44	CACNA2D1	c.659–2_659-1insT	NA	Female	14	ID	Yes (deeply set eyes, squared facies, bulbous nasal tip, full lips, horizontal eyebrows, enlarged nasal bridge, mouth with downward corners, anteverted nostrils)	Language delay, epilepsy
#45	GPR14 (before UTS2R)	c.844C > T	p.(Gln282 *)	Male	6	ID and ASD	Yes (long and large nose, broad nasal bridge, anteverted nostrils, deep philtrum)	Frequent infections

* means change in a stop codon.

## Data Availability

NGS data has been deposited in publicly accessible repositories. The data can be found here: http://nigdb.cineca.it/.

## Supplementary Materials

The following are available online at https://www.mdpi.com/article/10.3390/brainsci11070936/s1, Table S1: Clinical features of the 200 ID and ID/ASD patients; Table S2: P/LP variants identified in disease genes; Table S3: P/LP variants identified in candidate disease genes; Table S4: Uncertain variants identified by ES
