# Supplementary material for: Exome Sequencing in 200 Intellectual Disability/Autistic Patients: New Candidates and Atypical Presentations
Source: Brain Sci. 2021 Jul 16;11(7):936. doi: 10.3390/brainsci11070936 (PMC8303733; doi:10.3390/brainsci11070936)
Supplement: Supplementary file 1 [file brainsci-11-00936-s001.zip › Table S1.pdf]

Table S1. Clinical features of the 200 ID and ID/ASD patients.

Patient	Gender	Age (years)	ID/ASD	Craniofacial dysmorphisms	Additional clinical signs
**#1**	Male	7	ID	Yes (Plagiocephaly, prominent forehead, sparse eyebrows, broad nasal bridge, small ears, uplifted earlobes, conical teeth)	Bilateral clinodactyly of the 5th toe, supernumerary nipple, epilepsy, heteroaggressive behaviour
**#2**	Female	18	ID	Yes (microcephaly, triangular face, widow's peak, thick eyebrows, large nose, flat philtrum, thin lips, High vaulted and narrow palate)	Short stature, clinodactyly of the fifth finger, laryngomalacia, splenium thinning, epilepsy, hearing loss, flat foot, stereotypies, speech defect
**#3**	Female	22	ID	No	Epilepsy, ataxia, limited speech
**#4**	Male	14	ID and ASD	Yes (low hairline, periorbital swelling, big mouth, full lips)	Stereotypies
**#5**	Female	22	ID	Yes (elongated facies, prominent ears, high nasal bridge, open mouth and protruded tongue)	Dystonia, myoclonia, drug-resistant epilepsy, language delay, psychomotor delay, bruxism, hypertrichosis
**#6**	Female	16	ID	Yes (facies with coarse features, synophria, bulbous nose, large ears, large mouth)	Dystonia, myoclonia, hypertrichosis, language delay, drug-resistant epilepsy, psychomotor delay
**#7**	Male	14	ID	Yes (Craniosynostosis, long filter, long eyelashes, thin upper lip)	Stereotypies, alternating hemiplegia, language delay
**#8**	Female	20	ID	Yes (Open mouth, protruding tongue, narrow palate, dental anomalies)	Epilepsy, hypotonia, developmental delay, absent language
**#9**	Female	22	ID	Yes (broad eyebrows, low anterior and posterior hairline, forehead hypertrichosis, prominent ears, eye asymmetry, large nose with wide nostrils, large mouth, dental anomalies)	Upper limb hypotonia, lower limb hypertonia, recurrent infections, thinning corpus callosum, growth retardation, pectus excavatum, clinodactyly of the 5th fingers.

**#10**	Male	11	ID	Yes (low hairline, flattened nasal bridge, bulbous nasal tip, anteverted nostrils, full lips, small and widely spaced teeth, anteverted ears)	Epilepsy, language delay
**#11**	Female	12	ID and ASD	Yes (Microcephaly, long face, smooth and long philtrum, strabismus, upslanting palpebral fissures)	Developmental delay, stereotypic hand movements, hypotonia, bruxism, scialorrea, corpus callosum hypoplasia
**#12**	Male	16	ID	Yes (Microcephaly, narrow forehead, frontal bossing, depressed nasal bridge, short philtrum, prognathism)	Hypotonia, epilepsy, stereotypies, absent language
**#13**	Female	22	ID	Yes (prominent columella, sloping forehead, high and scattered eyebrows, deeply set eyes, nose with anteverted nostrils, narrow palate, prominent incisors, prominent ears)	Hypertrichosis, small hands and feet, joint hyperextensive
**#14**	Female	4	ID and ASD	Yes (craniosynostosis, flat forehead, downslanting palpebral fissures, high nasal bridge)	Language delay, hyperchromic and hypochromic spots
**#15**	Male	16	ID	Yes (proptosis, long eyelashes, synophria, narrow palate, dental anomalies, small mouth and full lips)	Bilateral cryptorchidism, psychomotor delay, poor vision, ventricular system dilation, immature hippocampal structures, spastic paraparesis, epilepsy, hypothonia, absent language
**#16**	Female	22	ID	No	Strabismus, cerebral atrophy, sialorrhea, motor problems, bruxism
**#17**	Female	6	ID	Yes (cleft palate, deeply set eyes, high nasal bridge, bulbous nasal tip)	Language delay, epilepsy, aggressiveness
**#18**	Male	39	ID and ASD	Yes (triangular facies, high nasal bridge, bulbous nasal tip, open mouth, micrognathia, narrow and downturned eyelids)	Stereotypies, aggressiveness, bladder anomalies
**#19**	Female	16	ID	No	Spastic paraparesis, behaviour disorder, slight enlargement of the interfolial spaces of the cerebellar hemispheres, hypertone

**#20**	Female	10	ID	Yes (sparse eyebrows, small nose, thin upper lip, dental anomalies, chubby cheeks)	Language delay, cerebellar and worm atrophy, psychomotor delay, brain abnormalities, hypertrichosis, bilateral clinodactyly of the second/third/fourth/fifth toes
**#21**	Male	49	ID	No	Ataxia, spastic paraparesis, angioma, nystagmus, epilepsy
**#22**	Male	16	ID	Yes (thick eyebrows, narrow and short eyelids, depressed helix, nose with bulbous tip, thick lips)	Hypertrichosis of the legs, arms and lumbar region, large hands, short fifth ray of the foot
**#23**	Female	2	ID	No	Motor stereotypies, epilepsy, thinning of the corpus callosum, ventricular enlargement
**#24**	Female	49	ID	Yes (sparse eyebrows, hypertelorism, narrow eyelids, gingival hypertrophy)	Syndactyly of the second and third toes, small feet, hypertrophy of the limbs and truncal obesity, strabismus
**#25**	Male	12	ID	Yes (synophria, horizontal eyebrows, wide nasal tip, anteverted nostrils, long filter, dental anomalies)	Spastic paraparesis, language delay, polyneuropathy
**#26**	Female	6	ID	Yes (microcephaly, deeply set eyes, nose with bulbous tip, anteverted nostrils, full lips)	Hyperactivity, blepharophimosis, brachydactyly, nail hypoplasia, kidney abnormalities, language delay
**#27**	Male	12	ID	Yes (narrow bitemporal diameter, narrow and upward eyelid rims, deep philtrum, progatism, exaggerated Cupid's bow, , buccal rim pointing downwards, uplifted ear lobe)	Hypotonia, obesity
**#28**	Female	44	ID	No	Microcephaly, brachydactyly, nail hypoplasia
**#29**	Male	2	ID	Yes (high forehead, low-set ears with large, downward-pointing auricles, down-slanting eyelids, broad nasal tip, long and thick filter, exaggerated Cupid's bow)	Café au lait spots in the thoracic and lumbar region, nail hypoplasia of the fifth toe, neurodevelopmental delay, cryptorchidism

**#30**	Male	18	ID	Yes (microcephaly, thick eyebrows, deeply set eyes, square chin)	Epilepsy, thinning corpus callosum, language delay, cold extremities, cerebellar atrophy, muscle hypotrophy
**#31**	Male	45	ID and ASD	Yes (alopecia in the fronto-temporal region, depressed ocular region, thin upper lip)	Absent language, obsessive behaviour, tapered fingers with widening of the intermediate interphalangeal joints, nail dystrophy, clinodactyly of the third toe, stereotypies, mouth chewing automatisms
**#32**	Male	21	ID and ASD	No	Epilepsy, psychomotor delay
**#33**	Male	18	ID	No	Language delay, psychomotor delay, manual stereotypies, increased tolerance to pain
**#34**	Female	10	ID	Yes (high forehead, spaced teeth, pursed lips attitude)	Epilepsy, neurodevelopmental regression, stereotypies, syndactyly
**#35**	Male	17	ID	Yes (sloping forehead, prognathism)	Strabismus, hyperchromic spot in the thoracic area, cerebellar atrophy, language delay, ataxia
**#36**	Female	26	ID and ASD	No	Stereotypies, self-harm, cold extremities, sphincter control not acquired, language regression
**#37**	Male	21	ID	Yes (bulbous nasal tip, full lips, spaced teeth)	Scoliosis, sialorrhoea, optic atrophy, psychomotor delay, epilepsy, spastic paraparesis, absent language.
**#38**	Male	10	ID and ASD	No	Gastroesophageal reflux, sphincter control not acquired, stereotyped behaviour
**#39**	Male	9	ID	Yes (advanced hairline, long and thick eyebrows, anteverted nostrils, long filter, thin upper lip)	Hypoplasia of the cerebellar vermis, thinned corpus callosum, cerebellar asymmetry, angioma, hypertrichosis, epilepsy, apraxia, ataxia and psychomotor delay

**#40**	Male	7	ID and ASD	Yes (Wide forehead, arched eyebrows, deeply set eyes, pointed chin)	Limited speech, neurodevelopmental delay
**#41**	Female	20	ID	Yes (thick eyebrows, prominent upper arch, hyperemic gums, high palate)	Limited speech, scoliosis, locomotor impairment, manual stereotypies, tapered fingers
**#42**	Male	6	ID and ASD	No	Language delay, oppositional and provocative behaviour
**#43**	Female	14	ID	Yes (microcephaly, synophria, long eyelashes, bulbous nasal tip)	Hypertrichosis, drug-resistant seizurs, spastic tetraparesis, renal failure.
**#44**	Female	14	ID	Yes (deeply set eyes, squared facies, bulbous nasal tip, full lips, horizontal eyebrows, enlarged nasal bridge, mouth with downward corners, anteverted nostrils)	Language delay, epilepsy,
**#45**	Male	6	ID and ASD	Yes (long and large nose, broad nasal bridge, anteverted nostrils, deep philtrum)	Frequent infections
**#46**	Male	15	ID	Yes (bulbous nasal tip, long eyelashes)	No
**#47**	Male	8	ID and ASD	No	Language delay, stereotypies, bruxism

**#48**	Male	9	ID and ASD	Yes (absent eyebrows, synophrys, epicanthic folds, bulbous nasal tip, long eyelashes)	Language delay, inverted nipples
**#49**	Male	18	ID and ASD	No	Language delay, manual stereotypies, psichomotor delay, short stature
**#50**	Male	16	ID and ASD	Yes (downslanting palpebral rims, synophrys)	Neurodevelopmental regression, epilepsy, bilateral sandal gap
**#51**	Male	15	ID	Yes (external eyelid rim out and up)	Sandal gap, stereotypies, neuro /splanchnocranium disproportion, flat feet.
**#52**	Male	6	ID	Yes (downslanting eyelid rims, broad nasal bridge, anteverted nostrils, long eyelashes, horizontal eyebrows, thin upper lip)	Bilateral sandal gap, liqueur spaces expansion, epilepsy
**#53**	Male	10	ID and ASD	Yes (microcephaly)	Language delay
**#54**	Male	6	ID and ASD	No	Language delay
**#55**	Female	9	ID and ASD	Yes (Deep set eyes, anteverted nostrils)	Sandal gap, motor stereotypies, cardiac abnormalities
**#56**	Male	42	ID	No	Spastic paraparesis, epilepsy, language delay
**#57**	Female	12	ID	No	Stereotypies, growth delay,respiratory infections, short stature, coloboma

**#58**	Female	7	ID	Yes (dysmorphic auricles, nose with widened base)	Psychomotor delay, hyperechogenic intestine
**#59**	Male	20	ID	No	Spastic tetraparesis, psychomotor delay
**#60**	Female	13	ID	Yes (widow's peak , long face, large ears)	No
**#61**	Male	5	ID and ASD	Yes (thin mouth, low set ears)	Clinodactyly, cerebellar lesion
**#62**	Male	15	ID	Yes (long facies, broad nasal bridge, narrow, advanced hairline on the forehead , piriform nose)	Diffuse muscle hypertrophy, hand stereotypies, leg varism,epilepsy, regression, language delay
**#63**	Male	15	ID and ASD	Yes (synophria)	Sandal gap, epilepsy
**#64**	Female	3	ID and ASD	Yes (hypertelorism, bulbous nasal tip, Cupid's bow)	Bruxism, language delay, neurodevelopmental delay, stereotypes,
**#65**	Female	12	ID	Yes (thick eyebrows, tubular nose, thin upper lip)	Stereotypies, bilateral sandal gap, growth delay, trunk-limbs disproportion, dysplasia of the hips
**#66**	Male	16	ID	Yes (microcephaly)	Psychomotor delay, language delay
**#67**	Male	21	ID and ASD	Yes (macrocephaly)	Overgrowth, periventricular leukomalacia
**#68**	Female	17	ID	No	Hypoacusia, language delay, psychomotor delay

**#69**	Male	15	ID	Yes (thick eyebrows, anteverted nostrils, hypertelorism, widened nasal bridge, folded helix, narrow palate)	Tapered fingers, brachydactyly
**#70**	Male	18	ID	No	Cerebellar atrophy, thinning corpus callosum, epilepsy, language delay, psychomotor delay
**#71**	Male	20	ID	Yes (high and narrow palate, irregularity of the helix of the ear, dental anomaly)	Tolerance for pain, spastic tetraparesis, epilepsy, developmental delay
**#72**	Female	28	ID	Yes (triangular facies, high nasal bridge, hypoplastic nose wings, mandibular hypoplasia, nystagmus)	Thyroid tissue agenesis, precocious puberty, hypertrichosis, tremor, muscular hypotrophy,
**#73**	Male	14	ID	Yes (deep-set eyes, anteverted nostrils, shortness of distal phalanx toes, microcephaly)	No
**#74**	Female	21	ID	Yes (microcephaly)	Truncal obesity
**#75**	Female	8	ID	Yes (microcephaly)	Low weight, epilepsy, developmental delay.
**#76**	Male	4	ID	No	Psychomotor retardation, hypotonia, bruxism
**#77**	Male	10	ID	No	Bruxism, stereotyped movements, psychomotor retardation
**#78**	Female	44	ID	Yes (prognathism)	Manual stereotypies along the midline, flat feet
**#79**	Female	10	ID	No	Psychomotor regression, poor motor development, absence of language

**#80**	Female	15	ID	Yes (elongated facies, high forehead, high nasal bridge, bulbous tip, hypoplastic nostrils)	No
**#81**	Female	13	ID	Yes (elongated eyelids, long eyelashes)	No
**#82**	Female	31	ID	No	Corpus callosum thickening
**#83**	Female	13	ID	Yes (microcephaly)	Epilepsy, absence of language
**#84**	Female	19	ID and ASD	No	Epilepsy, psychomotor delay
**#85**	Female	15	ID	Yes (dental anomalies , sinophris, eyelid rims up and out, high and narrow palate)	Corpus callosum abnormalities.
**#86**	Female	22	ID	No	Dystonia, myoclonia, drug-resistant epilepsy
**#87**	Female	23	ID and ASD	No	Manual stereotypies
**#88**	Female	10	ID and ASD	Yes (slightly protruding lobes, anteverted nostrils, spaced teeth)	Muscular hypotrophy, manual stereotypies, flat feet, clinodactyly of the III finger
**#89**	Female	15	ID and ASD	Yes (macrocephaly)	No
**#90**	Female	16	ID and ASD	No	Laryngomalacia, hypotonia, syndactyly,epilepsy

**#91**	Male	28	ID	No	Cryptorchidism, creatinine deficit
**#92**	Female	26	ID and ASD	No	Epilepsy, vesical anomalies, patent oval foramen
**#93**	Male	29	ID and ASD	No	Supernumerary nipples
**#94**	Male	31	ID and ASD	No	Macrocephaly, stains
**#95**	Male	16	ID and ASD	Yes (patent foramen ovale, hypochromic stain)	Hypotonia, bruxism, psychomotor delay
**#96**	Female	31	ID and ASD	Yes (macrocephaly)	Short stature, face and arms hypertrichosis, valgus hallux, obesity
**#97**	Male	15	ID and ASD	Yes (dental anomalies)	Language delay, poor visual contact, psychomotor delay, recurrent fever
**#98**	Male	18	ID and ASD	No	Hearing loss
**#99**	Male	9	ID and ASD	No	Language delay, hyperchromic stain, angioma
**#100**	Male	20	ID and ASD	Yes (micrognathia)	Absent language
**#101**	Female	16	ID and ASD	Yes (sinophris,nose with bulbous tip, hypoplastic nares, oval facies, deep eyes)	Obesity, ADHD, sandal gap

**#102**	Male	10	ID and ASD	No	Absent language, stereotypies, hypercholesterolemia
**#103**	Female	21	ID	Yes (thick eyebrows, eyes asymmetry, dental anomalies, large nose with wide nostrils)	Hypotonia, developmental delay, recurrent infection, corpus callosum thinning
**#104**	Male	9	ID and ASD	No	Cryptorchidism
**#105**	Female	16	ID and ASD	No	Epilepsy, ligamentous hyperlaxity
**#106**	Male	7	ID and ASD	No	Motor stereotypies, poor visual contact, bruxism, absent language, bronchospasm
**#107**	Male	9	ID and ASD	Yes (bulbous nasal tip, big eyes, big ears, micrognathia)	Motor stereotypies
**#108**	Female	13	ID	Yes (horizontal eyebrows, deep set eyes, anteverted nostrils, pointed chin)	Skeletal anomalies, fetal pads
**#109**	Female	5	ID	Yes (anteverted nostrils, broad nose root, long nasal filter)	Language delay, psychomotor delay, clinodactyly V finger
**#110**	Female	24	ID and ASD	Yes (microcephaly)	Spastic-dystonic tetraparesis, scoliosis, motor stereotypies, bruxism, sialorrhea.
**#111**	Male	12	ID and ASD	Yes (Mild hirsutism localized to the face, anteverted nostrils, triangular facies, large ears)	Motor stereotypies, psychomotor delay, epilepsy, language delay
**#112**	Female	4	ID	Yes (microcephaly, deeply set eyes)	Growth delay, language delay

**#113**	Female	5	ID	Yes (sparse eyebrows, long eyelashes, prominent ears)	Language delay
**#114**	Female	18	ID	No	Epilepsy, motor stereotypies
**#115**	Male	14	ID and ASD	No	No
**#116**	Female	6	ID and ASD	No	Language regression
**#117**	Male	12	ID	Yes (prominent ears, strabismus)	Psychomotor delay
**#118**	Male	10	ID and ASD	Yes (high hairline front, broad forehead, deeply set eyes, large mouth, macrostoma, lateral cleft)	No
**#119**	Female	6	ID and ASD	No	No
**#120**	Male	26	ID and ASD	No	Epilepsy
**#121**	Male	9	ID and ASD	Yes (tubular nose, elongated face, hypotelorism, thin lips)	Epilepsy, language delay
**#122**	Male	10	ID and ASD	Yes (Broad forehead, anteverted nostrils, dental anomalies, uplifted earlobes)	Motor stereotypies
**#123**	Female	9	ID and ASD	No	Language regression

**#124**	Male	9	ID and ASD	Yes (thick eyebrows thin upper lip and thick lower lip, deeply set eyes)	Motor stereotypies, language delay
**#125**	Male	5	ID and ASD	Yes (large nose, Broad nasal bridge, Anteverted nostrils, Flat philtrum)	Language regression
**#126**	Male	17	ID and ASD	No	Language delay, psychomotor delay
**#127**	Male	6	ID and ASD	Yes (Macrocephaly)	No
**#128**	Male	5	ID and ASD	Yes (Lateral thinning of eyebrows, broad nasal bridge, middle face hypoplasia, epicanthic fold, long eyelashes)	Syndactyly, language delay
**#129**	Female	9	ID	No	Motor stereotypies
**#130**	Male	6	ID and ASD	No	Hyperactivity, language delay, psychomotor delay
**#131**	Female	9	ID	Yes (Deep set eyes, High hairline front, broad nasal root)	syndactyly of the II-III toe, psychomotor delay
**#132**	Female	5	ID and ASD	No	Motor stereotypies, language delay
**#133**	Female	5	ID and ASD	Yes (long eyelashes, anteverted nostrils, short philtrum, everted lip)	Hypertrichosis, motor stereotypies
**#134**	Female	8	ID	Yes (Deep set eyes, tubular nose, small mouth with thin lips)	Behaviour disorder
**#135**	Male	5	ID and ASD	Yes (upslanting of palpebral fissures, wide mouth, spaced teeth)	Absent language, stereotypies

**#136**	Male	5	ID and ASD	No	Absent language
**#137**	Female	20	ID and ASD	No	Obesity
**#138**	Male	8	ID and ASD	Yes (bulbous nasal tip, deeply set eyes, Cupid's bow)	Ligamentous laxity
**#139**	Male	8	ID and ASD	Yes (Synophrys, Prognathism)	Hypertrichosis
**#140**	Male	7	ID	Yes (prominent columella, thin upper lip)	Language delay
**#141**	Female	7	ID and ASD	No	Hyperactivity, psychomotor delay
**#142**	Female	21	ID and ASD	Yes (low frontal hairline, thick eyebrows, broad nasal bridge with bulbous tip, widened nostrils, large mouth, thick lower lip, dental anomalies, dystrophic nails)	Language delay, hypoplasia of the corpus callosum, scoliosis, hypertrichosis
**#143**	Male	11	ID	Yes (small forehead, thick eyebrows, narrow eyelids, thin upper lip, cleft lip , prognathism, prominent ears)	Muscle hypotonia
**#144**	Female	35	ID	No	Epilepsy
**#145**	Male	28	ID	No	No
**#146**	Male	11	ID	No	Epilepsy, motor stereotypies

**#147**	Female	8	ID and ASD	No	Absent language, stereotypies, psychomotor delay
**#148**	Male	8	ID	No	Epilepsy, psychomotor delay
**#149**	Male	6	ID and ASD	Yes (horizontal eyebrows, anteverted nostrils, cupid's bow, elongated eyelid rims)	Hyperactivity, psychomotor delay
**#150**	Female	8	ID and ASD	Yes (wide forehead, deeply set eyes, short nose, anteverted nostrils, long filter, thin lips)	Language delay, psychomotor delay
**#151**	Male	9	ID	Yes (deeply set eyes, tubular nose, thin upper lip, pointed palate, dental abnormalities)	Hyperactivity, behaviour disorder
**#152**	Male	18	ID and ASD	Yes (strabismus)	Repetitive movements, absent language
**#153**	Male	22	ID and ASD	No	Anxiety disorder
**#154**	Male	8	ID and ASD	No	Repetitive movements, psychomotor delay
**#155**	Male	21	ID and ASD	Yes (face telangiectasias)	Psychomotor retardation, behaviour disorder, hypercholesterolemia, hyperactivity
**#156**	Male	11	ID and ASD	No	Epilepsy, psychomotor retardation
**#157**	Male	7	ID and ASD	No	Behaviour disorder,, motor stereotypies

**#158**	Male	18	ID and ASD	Yes (Horizontal eyebrows, synophria, low-set ears.)	Psychomotor retardation, language delay
**#159**	Male	14	ID and ASD	No	Repetitive behaviors, manual stereotypies, episodes of hetero aggression, psychomotor delay
**#160**	Male	9	ID and ASD	No	Language delay, stereotypies.
**#161**	Male	12	ID and ASD	No	Language delay
**#162**	Male	16	ID and ASD	No	Neurodevelopmental regression, stereotypes.
**#163**	Male	21	ID	No	Developmental delay, unmotivated laughter and crying.
**#164**	Male	30	ID and ASD	No	Manual and vocal stereotypies.
**#165**	Male	27	ID and ASD	No	No
**#166**	Male	28	ID and ASD	No	No
**#167**	Male	7	ID	No	Psychomotor delay
**#168**	Male	8	ID and ASD	Yes (small auricles, retrognathia)	Language delay, bilateral syndactyly II and III toes
**#169**	Male	19	ID and ASD	No	Language delay, self-harm, bruxism.

**#170**	Male	13	ID and ASD	No	Language delay, social isolation, hyperphagia. self-harm.
**#171**	Male	8	ID and ASD	No	Language delay, social isolation
**#172**	Female	8	ID	No	Language delay, stereotypies, epilepsy
**#173**	Male	12	ID and ASD	No	Syndactyly II-III toe, hyperactivity.
**#174**	Male	12	ID and ASD	No	No
**#175**	Male	12	ID and ASD	No	No
**#176**	Male	9	ID and ASD	No	No
**#177**	Male	9	ID and ASD	No	No
**#178**	Male	12	ID	No	Language delay, stereotypies
**#179**	Male	15	ID and ASD	No	No
**#180**	Male	15	ID and ASD	No	No

**#181**	Male	18	ID and ASD	No	Language delay, episodes of hetero aggression, stereotypies
**#182**	Male	15	ID and ASD	No	Absent language, stereotypies
**#183**	Male	23	ID and ASD	No	Language delay, seizures
**#184**	Male	11	ID and ASD	Yes (Low frontal hairline, thick eyebrows, long eyelashes, deeply set eyes, nose with bulbous tip, thick columella and hypoplastic nostrils, auricles with uplifted lobe)	Language delay
**#185**	Male	9	ID and ASD	No	Language delay, stereotypies, apraxia
**#186**	Male	18	ID and ASD	No	No
**#187**	Male	8	ID and ASD	No	Absent language, stereotypies
**#188**	Male	13	ID and ASD	No	Hyperactivity, aortic coarctation
**#189**	Male	10	ID and ASD	No	Motor stereotypies, aggressiveness
**#190**	Male	9	ID and ASD	Yes (bulbous nasal tip, broad bridge, long and narrow eyelids, high forehead)	Language delay

**#191**	Male	9	ID and ASD	Yes (flat occipitus)	No
**#192**	Male	8	ID and ASD	Yes (anteverted nostrils)	Motor stereotypies, psychomotor regression, hyperactivity, language delay, clinodactyly of the V toe
**#193**	Male	9	ID and ASD	Yes (scattered eyebrows, bulbous nasal tip , large eyes)	Language regression
**#194**	Male	9	ID and ASD	Yes (macrocephaly)	Language delay, motor stereotypies
**#195**	Male	8	ID and ASD	No	Manual stereotypies, language delay, psychomotor delay
**#196**	Male	9	ID and ASD	No	Language delay, psychomotor delay
**#197**	Male	9	ID and ASD	No	Repetitive movements
**#198**	Male	19	ID and ASD	No	Language delay, hyperactivity, recurrent infections
**#199**	Female	10	ID and ASD	No	Psychomotor delay, language delay, repetitive movements
**#200**	Male	9	ID and ASD	No	Clinodactyly of the IV toe
