# Supplementary material for: Exome Sequencing in 200 Intellectual Disability/Autistic Patients: New Candidates and Atypical Presentations
Source: Brain Sci. 2021 Jul 16;11(7):936. doi: 10.3390/brainsci11070936 (PMC8303733; doi:10.3390/brainsci11070936)
Supplement: Supplementary file 1 [file brainsci-11-00936-s001.zip › Table S2.pdf]

Table S2. P/LP variants identified in disease genes.

Patient	Gene	Status	Transcript (hg19)	genomic DNA position	Variant (HGVS)	Protein (HGVS)	Location (Exon /Intron)	Variant effect	dbSNP	MAF (gnomAD %)	CADD	origin	ClinVar	References
**#1**	*ADNP*	heterozygous	NM_015339.2	20:49508937	c.2314dup	(p.(Thr772Asnfs*16)	5	frameshift	NR	NR	NA	ND	NR	NR
**#2**	*AP4M1*	heterozygous	NM_004722.3	7:99704400_99704425del	c.1257_1282del	p.(Val421Alafs*98)	15	frameshift	rs977969791	NR	NA	inherited	NR	NR
**#2**	*AP4M1*	heterozygous	NM_004722.3	7:99704460	c.1317G>A	p.(Trp439*)	15	nonsense	rs772119268	0.00040 %	NA	inherited	NR	NR
**#3**	*ATP1A3*	heterozygous	NM_152296.4	19:42479928	c.2116G>A	p.(Gly706Arg)	16	missense	rs782175860	NR	25.1	de novo	Pathogenic	[Hully et al., 2017]
**#4**	*BCOR*	hemizygosis	NM_017745.5	X: 39933823	c.776C>T	p.(Ser259Leu)	4	missense	rs727503826	0 %	25.3	de novo	Pathogenic	NR
**#5 #6**	*BSCL2*	homozygous	NM_032667.6	11:62458571	c.856C>T	p.(Arg286*)	8	nonsense	rs763070770	0.0016 %	NA	inherited	NR	NR
**#7**	*CACNA1A*	heterozygous	NM_000068.3	19:13373594	c.4055G>A	p.(Arg1352Gln)	25	missense	rs1057520918	NR	32	de novo	Pathogenic	[Stubberudet al., 2021]
**#8**	*CSNK2B*	heterozygous	NM_001320.6	6:31635742	c.170del	p.(Glu57Glyfs*15)	3	frameshift	NR	NR	NA	de novo	NR	NR
**#9**	*CTU2*	heterozygous	NM_001012759.2	16:88780062	c.881C>A	p.(Ser294*)	9	nonsense	rs201111272	0.019 %	NA	inherited	NR	NR
**#10**	*DEPDC5*	heterozygous	NM_001242896.1	22:32210985	c.1453C>T	p.(Arg485*)	21	nonsense	rs1568991466	NR	NA	de novo	Pathogenic	NR
**#11**	*DDX3X*	heterozygous	NM_001356.4	X:41203603	c.976C>T	p.(Arg326Cys)	10	missense	rs1555953548	NR	32	de novo	Pathogenic	NR

**#12**	*DYRK1A*	heterozygous	NM_001396.3	21:38878524	c.1669C>T	p.(Gln557*)	10	nonsense	NR	NR	NA	de novo	NR	NR
**#13**	*EFTUD2*	heterozygous	NM_004247.3	17:42956923	c.702+1G>A	NA	9	splicing	NR	NR	NA	de novo	NR	NR
**#14**	*FGFR3*	heterozygous	NM_000142.4	4:1803571	c.749C>G	p.(Pro250Arg)	7	missense	rs4647924	0.00074 %	22.1	de novo	Pathogenic /Likely pathogenic	[Aravidis et al., 2014]
**#15**	*HK1*	heterozygous	NM_033496.2	10:71142347	c.1367C>T	p.(Thr456Met)	10	missense	rs1057517928	NR	31	de novo	Pathogenic /Likely pathogenic	[Okur et al., 2019]
**#16**	*IQSEC2*	heterozygous	NM_001111125 .2	X:53264088	c.3780del	p.(Gln1261Serf s*136)	15	frameshift	NR	NR	NA	de novo	NR	NR
**#17**	*KANSL1*	heterozygous	NM_001193466 .1	17:44248524_442 48525del	c.985_986del	p.(Leu329Glufs *22)	2	frameshift	rs281865473	0.0028 %	NA	de novo	Pathogenic/ Uncertain significance	[Koolen et al.,2016]
**#18**	*KCNQ3*	heterozygous	NM_004519.3	8: 133192493	c.688C>T	p.(Arg230Cys)	5	missense	rs796052676	NR	31	de novo	Pathogenic/ Likely pathogenic	NR
**#19**	*KIF1A*	heterozygous	NM_001244008 .1	2:241737133	c.37C>T	p.(Arg13Cys)	1	missense	rs1064794935	NR	32	de novo	Likely pathogenic/ Pathogenic/ Uncertain significance	[Kurihara et al.,2020]
**#20** **#21**	*KIF1A*	heterozygous	NM_001244008 .1	2:241715312	c.914C>T	p.(Pro305Leu)	11	missense	rs1131690804	NR	27.4	de novo	Pathogenic/ Likely pathogenic	[Spagnoli et al., 2019]
**#22**	*KMT2A*	heterozygous	NM_001197104 .1	11:118365075	c.5256del	p.(Ala1753Prof s*70)	17	frameshift	NR	NR	NA	de novo	NR	NR
**#23**	*MBOAT7*	heterozygous	NM_024298.4	19:54687420	c.477C>G	p.(Tyr159*)	5	nonsense	NR	NR	NA	inherited	NR	NR
**#24**	*MED13L*	heterozygous	NM_015335.4	12:116714864	c.72+1G>T	NA	1	splicing	NR	NR	NA	de novo	NR	NR

**#25**	*MMACHC*	homozygous	NM_015506.2	1:45974478	c.440G>C	p.(Gly147Ala)	4	missense	rs140522266	0.032 %	24	inherited	Pathogenic/ Likely pathogenic	NR
**#26 #27 #28**	*POGZ*	heterozygous	NM_015100.3	1:151397435_151397436del	c.1180_1181del	p.(Met394Valfs*9)	8	frameshift	rs1057518170	NR	NA	inherited	Pathogenic	NR
**#29**	*PTPN11*	heterozygous	NM_002834.3	12:112926851	c.1471C>A	p.(Pro491Thr)	13	missense	rs397507539	NR	23.1	de novo	Pathogenic/ Likely pathogenic	NR
**#30**	*RHOBTB2*	heterozygous	NM_015178.2	8:22865140	c.1382G>A	p.(Arg461His)	9	missense	rs1554504663	NR	29.1	de novo	Pathogenic/ Likely pathogenic	NR
**#31**	*SHANK3*	heterozygous	NM_001080420.1	22:51158930_51158931dup	c.2717_2718dup	p.(Ser907Alafs*3)	21	frameshift	NR	NR	NA	de novo	NR	NR
**#32**	*SHANK3*	heterozygous	NM_033517.1	22:51153476	c.2313+1G>A	NA	19	splicing	rs1396379503	0.0012 %	NA	de novo	Pathogenic/ Likely pathogenic	NR
**#33**	*SHANK3*	heterozygous	NM_001080420.1	22:51159463_51159466del	c.3250_3253del	p.(Leu1084Cysfs*9)	22	frameshift	NR	NR	NA	de novo	NR	NR
**#34**	*SPG7*	heterozygous	NM_003119.2	20:89576947	c.233T>A	p.(Leu78*)	2	nonsense	rs121918358	0.040 %	NA	inherited	Pathogenic	[Balicza et al., 2016]
**#35**	*SPTBN2*	heterozygous	NM_006946.2	11:66475652	c.1310G>A	p.(Arg437Gln)	11	missense	NR	NR	28.8	ND	NR	[Accogli et al., 2020]
**#36**	*SYNGAP1*	heterozygous	NM_006772.2	6:33410665	c.2337-1G>A	NA	14	splicing	NR	NR	NA	de novo	NR	NR
**#37**	*TBCE*	heterozygous	NM_001079515.1	1:235564851dup	c.134dup	p.(Arg46Glufs*5)	3	frameshift	NR	NR	NA	de novo	NR	NR
**#37**	*TBCE*	homozygous	NM_001079515.1	1:235590458	c.464T>A	p.(Ile155Asn)	6	missense	rs780472451	0 %	25.6	inherited	Pathogenic	[Sferra et al., 2016]
**#38**	*TREX1*	heterozygous	NM_016381.3	3:48508447_48508462del	c.558_573del	p.(Phe186Leufs*24)	1	frameshift	NR	NR	NA	inherited	NR	NR

**#39**	*TUBA1A*	heterozygous	NM_001270399.1	12:49580116	c.352G>A	p.(Val118Met)	3	missense	rs863224938	NR	26.2	de novo	Pathogenic/ Likely pathogenic	NR
**#40**	*UPF3B*	hemizygosis	NM_080632.2	X:118971734	c.1288C>T	p.(Arg430*)	10	nonsense	rs122468181	NR	NA	inherited	Likely pathogenic	NR
**#41**	*WDR45*	heterozygous	NM_001029896.1	X:48935560	c.66del	p.(Cys23Alafs*15)	5	frameshift	NR	NR	NA	de novo	NR	NR
**#42**	*WFS1*	heterozygous	NM_006005.3	4:6279306	c.124C>T	p.(Arg42*)	2	nonsense	rs71530923	0.0097 %	NA	ND	Pathogenic/ Likely pathogenic	NR
**#43**	*WFS1*	heterozygous	NM_006005.3	4:6302752_6302755del	c.1230_1233del	p.(Leu412Serfs*29)	8	frameshift	rs760337383	0.0035 %	NA	ND	Pathogenic	NR

HGVS, Human Genome Variation Society (http://www.hgvs.org) ; ClinVar Clinical Variation database (https://www.ncbi.nlm.nih.gov/ClinVar/); MAF (Minor Allele Frequency); CADD (Combined Annotation Dependent Depletion); NA, not applicable; ND, not determined; NR, not reported
