# Supplementary material for: Exome Sequencing in 200 Intellectual Disability/Autistic Patients: New Candidates and Atypical Presentations
Source: Brain Sci. 2021 Jul 16;11(7):936. doi: 10.3390/brainsci11070936 (PMC8303733; doi:10.3390/brainsci11070936)
Supplement: Supplementary file 1 [file brainsci-11-00936-s001.zip › Table S3.pdf]

Table S3. P/LP variants identified in candidate disease genes.

Patient	Gene	Status	Transcript (hg19)	Genomic DNA position	Variant (HGVS)	Protein (HGVS)	Location (Exon/ Intron)	Variant effect	dbSNP	MAF (gnomad %)	origin
**#44**	*CACNA2D1*	heterozygous	NM_000722.2	7:81695840_81695841insT	c.659-1_659insA	NA	7	splicing	NR	NR	de novo
**#45**	*GPR14*	heterozygous	NM_018949.3	17:80333044	c.844C>T	p.(Gln282*)	3	nonsense	rs577182190	0.0023 %	de novo

HGVS, Human Genome Variation Society (http://www.hgvs.org); MAF (Minor Allele Frequency); NA, not applicable; NR, not reported.
