# Supplementary material for: Exome Sequencing in 200 Intellectual Disability/Autistic Patients: New Candidates and Atypical Presentations
Source: Brain Sci. 2021 Jul 16;11(7):936. doi: 10.3390/brainsci11070936 (PMC8303733; doi:10.3390/brainsci11070936)
Supplement: Supplementary file 1 [file brainsci-11-00936-s001.zip › Table S4.pdf]

Tabel S4. Uncertain variants identified by ES.

Patient	Gene	Transrip‍t (hg19)	Genomic DNA position	Variant (HGVS)	Protein (HGVS)	Location (Exon /Intron)	Variant effect	dbSNP	MAF (gnomAD%)	Origin	CADD	ClinVar
**#196**	*ACSL4*	NM_022977.2	X:108925954	c.523T>G	p.(Phe175Val)	5	missense	NR	NR	inherited	24.3	NR
**#122**	*AFF2*	NM_001169123.1	X:148037970	c.2365A>G	p.(Lys799Glu)	12	missense	rs782610217	0.00055 %	ND	25,00	NR
**#50**	*AFF2*	NM_002025.3	X:148048468	c.3062C>T	p.(Thr1021Ile)	15	missense	NR	0.00055 %	de novo	23.5	NR
**#147**	*ANK3*	NM_020987.3	10:61834572	c.6067G>A	p.(Ala2023Thr)	37	missense	rs146929899	0.028 %	de novo	14.65	uncertain significance
**#83**	*ARID1B*	NM_001346813.1	6:157099307	c.244C>A	p.(Gln82Lys)	1	missense	rs1460103880	0.0021 %	inherited	22.8	NR
**#150**	*ARID1B*	NM_001346813.1	6:157495210	c.3055G>A	p.(Gly1019Ser)	10	missense	rs375023508	0.0018%	de novo	24.5	NR
**#65**	*ATRX*	NM_000489.3	X:76875864	c.5271G>T	p.(Glu1757Asp)	20	missense	NR	NR	de novo	23.8	NR
**#52**	*ATRX*	NM_000489.3	X:76855024	c.5812A>G	p.(Lys1938Glu)	25	missense	NR	NR	de novo	19.48	NR
**#196**	*BCORL1*	NM_001184772.2	X:129154967	c.3449A>G	p.(Lys1150Arg)	4	missense	NR	NR	inherited	16.4	NR
**#172**	*CABLES1*	NM_001100619.2	18:20716361	c.635A>G	p.(Asp212Gly)	1	missense	NR	NR	de novo	24.2	NR
**#109**	*CACNA1A*	NM_023035.2	19:13409455	c.3004G>A	p.(Gly1002Arg)	19	missense	rs781006387	0.0025 %	inherited	23.1	NR

**#60**	*CACNA1G*	NM_018896.4	17:48639046	c.226C>A	p.(Arg76Ser)	1	missense	NR	NR	de novo	23.9	NR
**#120**	*CACNA1H*	NM_021098.2	16:1250375	c.923G>A	p.(Arg308His)	7	missense	rs1332969846	0.00039 %	de novo	22.9	NR
**#183**	*CACNA1H*	NM_021098.2	16:1259249	c.3581G>A	p.(Arg1194Gln)	17	missense	rs759668583	0.0058 %	inherited	19.07	uncertain significance
**#126**	*CAPRIN1*	NM_005898.4	11:34104424	c.879G>A	p.(Glu293=)	8	potentially splicing	NR	NR	de novo	25.4	NR
**#32**	*CENPJ*	NM_018451.4	13:25484137	c.656C>T	p.(Pro219Leu)	4	missense	rs139844197	0.046 %	inherited	14.6	uncertain significance
**#32**	*CENPJ*	NM_018451.4	13:25480327	c.1849C>T	p.(Pro617Ser)	7	missense	rs573822147	0.026 %	inherited	26.4	NR
**#74**	*CSNK2A1*	NM_177559.2	20:472938	c.581C>A	p.(Ser194Tyr)	9	missense	NR	NR	de novo	28.7	NR
**#9**	*CTU2*	NM_001012759.2	16:88779836	c.854C>A	p.(Ala285Asp)	8	missense	rs780088546	0.00079 %	inherited	31,00	NR
**#110**	*DEAF1*	NM_021008.3	11:686911	c.751G>A	p.(Asp251Asn)	5	missense	NR	NR	de novo	26.6	NR
**#123**	*DNM1L*	NM_012062.4	12:32861094	c.305C>T	p.(Thr102Met)	4	missense	rs201929226	0.028 %	de novo	25,00	uncertain significance
**#70**	*FGD1*	NM_004463.2	X:54496520	c.1030G>A	p.(Asp344Asn)	4	missense	rs781588464	0.0011 %	inherited	22.8	NR
**#131**	*GRIN1*	NM_000832.6	9:140056963	c.1859G>A	p.(Gly620Glu)	14	missense	NR	NR	de novo	25.9	NR

**#72**	*HUWE1*	NM_031407.5	X:53631745	c.2547C>G	p.(Asp849Glu)	26	missense	NR	NR	inherited	18.36	NR
**#34**	*MECP2*	NM_001110792.1	X:153296857	c.458A>C	p.(Tyr153Ser)	4	missense	NR	NR	de novo	26.4	NR
**#139**	*MED12*	NM_005120.2	X: 70360696-70360988del	c.6256_6258del	p.(Gln2086del)	42	inframe deletion	rs1246803647	0.021 %	de novo	NA	NR
**#59**	*MYCN*	NM_001293228.1	2:16082569	c.383G>A	p.(Arg128His)	2	missense	rs771914958	0.0012 %	de novo	31,00	NR
**#143**	*NBEA*	NM_015678.4	13:36239272	c.8350G>T	p.(Val2784Phe)	56	missense	rs41292207	0.27 %	de novo	26.2	NR
**#53**	*PHF6*	NM_001015877.1	X:133512133	c.237G>C	p.(Lys79Asn)	3	missense	NR	NR	inherited	25,00	NR
**#163**	*PLCL2*	NM_015184.5	3:17131351	c.2953C>T	p.(Arg985Cys)	6	missense	rs753953891	0.0008 %	de novo	31,00	NR
**#60**	*PTCHD1*	NM_173495.2	X:23398227	c.871G>A	p.(Asp291Asn)	2	missense	rs1341244854	0.00055 %	inherited	24.6	NR
**#151**	*RBMX*	NM_002139.3	X: 135956411	c.1066G>T	p.(Gly356Trp)	9	missense	rs35085326	0.92 %	de novo	26.5	NR
**#114**	*SCN2A*	NM_001040143.2	2:166234150	c.4298A>T	p.(Asp1433Val)	23	missense	NR	NR	de novo	25.6	NR
**#153**	*SCN2A*	NM_001040143.2	2:166226780	c.3820T>G	p.(Trp1274Gly)	20	missense	NR	NR	de novo	31,00	NR
**#66**	*SCN11A*	NM_001349253.1	3:38892017	c.4282G>A	p.(Gly1428Ser)	29	missense	rs201336927	0.020 %	inherited	29.2	uncertain significance

**#54**	*SHANK3*	NM_001080420.1	22:51121780	c.898C>T	p.(Arg300Cys)	8	missense	rs376862893	0.0097 %	de novo	31,00	NR
**#70**	*SLC9A7*	NM_032591.2	X:46502684	c.1600T>C	p.(Trp534Arg)	12	missense	rs138701264	0.026 %	inherited	28.1	NR
**#166**	*SLC12A2*	NM_001046.2	5:127503529	c.2693G>A	p.(Arg898Lys)	18	missense	rs762787955	0.00041 %	de novo	20.5	NR
**#186**	*SOX3*	NM_005634.2	X:139587213	c.13C>G	p.(Arg5Gly)	1	missense	NR	NR	inherited	15.88	NR
**#167**	*TMLHE*	NM_018196.3	X:154754267	c.208C>T	p.(Arg70Cys)	3	missense	rs200585049	0.030 %	inherited	24.2	NR
**#77**	*TH*	NM_000360.3	11:2185521	c.1436T>A	p.(Leu479Gln)	14	missense	NR	NR	inherited	24.8	NR
**#77**	*TH*	NM_000360.3	11:2189316	c.576+5G>C	NA	5	potentially splicing	NR	NR	inherited	NA	NR
**#67**	*TRIO*	NM_007118.3	5:14479388	c.6172T>A	p.(Tyr2058Asn)	42	missense	NR	NR	de novo	29.9	NR
**#56**	*ZNF462*	NM_021224.5	9:109689704	c.3511G>C	p.(Ala1171Pro)	3	missense	NR	NR	de novo	20.7	NR
**#148**	*ZNF711*	NM_021998.4	X:84502643	c.65T>C	p.(Ile22Thr)	3	missense	NR	NR	inherited	6.8	NR

HGVS, Human Genome Variation Society (http://www.hgvs.org) ; ClinVar Clinical Variation database (https://www.ncbi.nlm.nih.gov/ClinVar/); MAF (Minor Allele Frequency); CADD (Combined Annotation Dependent Depletion); NA, not applicable; ND, not determined; NR, not reported
